# Arch Length Discrepancy Analysis Comparison with the Huckaba and Moyers Method in Angle Class I Malocclusion in Surabayan Children with Mixed Dentition during the Growth and Development Phase: A Retrospective Cross-Sectional Study in Universitas Airlangga Dental Hospital

**DOI:** 10.1055/s-0045-1807732

**Published:** 2025-05-01

**Authors:** Alexander Patera Nugraha, Delfia Amanda Putri, Andree Salim, Ida Bagus Narmada, Ari Triwardhani, I. Gusti Aju Wahju Ardani, Alida Alida, Adya Pramusita, Tengku Natasha Eleena Binti Tengku Ahmad Noor

**Affiliations:** 1 Department of Orthodontics, Faculty of Dental Medicine, Airlangga University, Surabaya, Indonesia; 2 Faculty of Dental Surgery, Royal College of Surgeon, Edinburgh University, United Kingdom; 3 Malaysian Armed Forces Dental Officer, 609 Armed Forces Dental Clinic, Kem Semenggo, Kuching, Serawak, Malaysia

**Keywords:** mixed dentition analysis, malocclusion, discrepancy, Huckaba method, Moyers method

## Abstract

**Objective:**

The aim of the study was to compare the ALD results obtained using the Huckaba and Moyers methods, including the segmentation and brass wire techniques, in class I Angle malocclusion in Surabayan children during the MD and GDP at Dental Hospital Universitas Airlangga.

**Materials and Methods:**

This study adopted a retrospective cross-sectional design, involving a total of 252 samples. However, only 32 samples that met the inclusion criteria were analyzed for ALD by using the Huckaba and Moyers methods. Sex, chronological age, and malocclusion angle classification were recorded. Cervical maturation vertebrae stage was conducted to determine the skeletal age, while dental age was investigated through the Demirjian method in the GDP during MD in Javanese children. Lateral cephalogram was examined to determine skeletal malocclusion based on the Steiner analysis (Sella-Nasion-A [<SNA], Sella, Nasion, B [<SNB], [SNA-SNB] <ANB).

**Statistical Analysis:**

The Shapiro–Wilk and Levene tests revealed nonnormal distribution and lack of homogeneity, leading to the use of nonparametric Kruskal–Wallis and Mann–Whitney U tests for identifying significant differences.

**Results:**

In the upper and jaw, the Moyers method demonstrated a positive ALD value, whereas the Huckaba method exhibited a negative ALD value, indicating a significant difference between the groups.

**Conclusion:**

Discrepancy analysis revealed significant differences between the Huckaba and Moyers methods for class I Angle malocclusion in Surabayan children during MD and GDP at Dental Hospital Universitas Airlangga.

## Introduction

As individuals age, they experience significant dentocraniofacial changes, including changes in dental arch and tooth alignment. 1 In children still in the growth and development phase (GDP), both primary and permanent teeth can be present in the oral cavity, a stage known as mixed dentition (MD). 2 One common issue during this transitional dentition phase is the mismatch between arch length and tooth size, which can lead to malocclusion. 3 According to World Oral Health, malocclusion is the third most common oral health issue, affecting function, neuromuscular health, and aesthetics. 4

MD analysis aids in diagnosing and managing malocclusion by assessing the discrepancy between available and required spaces during the transitional dentition phase, known as arch length discrepancy (ALD) analysis. 5 6 The Moyers method is favored for its simplicity and nonreliance on radiographs, while the Huckaba method utilizes orthopantomogram (OPG) radiographic measurements for accuracy. 7 8 Both methods were originally developed for the Caucasian populations, highlighting the need for adaptation in diverse groups. 9 10

Various methods for measuring the dental arch perimeter are used to determine the available space, including the segmented technique and the use of brass wire used in the Moyers method. 11 In the segmented technique, although the method is simple, reducing the rounded arch perimeter into straight-line segments provides a lower estimate. On the other hand, while the brass wire method follows the shape of the basal arch, it is more time-consuming than the segmented technique and has lower reproducibility. 12 13 14 Both techniques need to be compared, as there is no standardized approach for measuring ALD.

Prior to this study, no research has specifically compared the Moyers and Huckaba methods using brass wire and segmentation techniques applied to non-Caucasian populations. However, to date, studies comparing the Huckaba and Moyers methods remain limited, especially in the Surabayan Deutromalay ethnic subgroup. Therefore, this study was conducted to compare these methods for application in Angle class I malocclusion cases among Surabayan children in the MD and GDP at the Dental Hospital Universitas Airlangga Surabaya.

## Materials and Methods

### Study Design and Ethical Clearance

This study adopted a retrospective cross-sectional design, involving a population of 252 patients from the Orthodontics Department at the Dental Hospital, Universitas Airlangga Surabaya, conducted between July and November 2024. The population comprised children aged 7 to 12 years who had either never received or previously undergone orthodontic treatment. The study utilized OPG, lateral cephalogram, and study models collected from orthodontic patients treated between 2022 and 2024. The research protocol received ethical approval from the Health Research Ethics Committee, Faculty of Dental Medicine, Universitas Airlangga (0931/HRECC.FODM/VIII/2024).

**Fig. 1:**
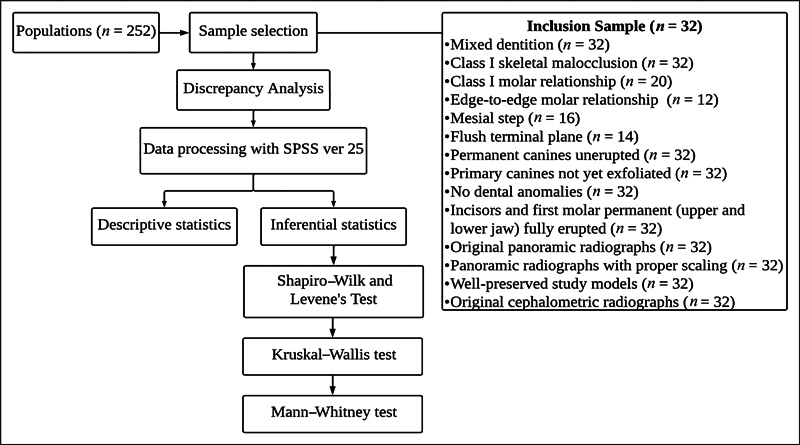
Sample selection process based on the inclusion and exclusion criteria.

From 252 patients, 32 samples were selected based on the inclusion criteria. The inclusion criteria were skeletal class I malocclusion, class I molar relationship or edge-to-edge relation with the second primary molar in a flush terminal plane or mesial step relationship, fully erupted first permanent molars, permanent lateral and central incisors in all arches, an OPG with a definitive scale with original film, and original lateral cephalogram images. Samples with erupted permanent canines, agenesis of permanent canines and premolars, premature loss of primary canines, congenital defects, and tooth deformities such as macrodontia and microdontia were excluded (see Fig. 1 ).

**Fig. 2:**
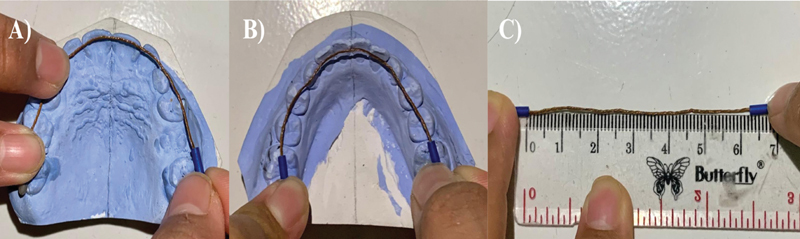
Brass wire technique. ( **A** ) Upper jaw. ( **B** ) Lower jaw. ( **C** ) Measuring with a ruler as the research instruments used in this study.

The discrepancy value is obtained by subtracting the required space from the available space. The available space calculation was performed using two techniques: the brass wire technique and the segmentation technique. In the brass wire technique, this study used a 0.5-mm brass wire. The method involves extending the brass wire from the mesiobuccal cusp of the first permanent molar on one side to the mesiobuccal cusp of the molar on the opposite side. The wire passes over the buccal prominence for the lower cast (see Fig. 2A ) or through the external fissure of the upper jaw (see Fig. 2B ), and aligns with the incisal edges of the teeth to form the correct curve. The wire is then straightened, and its length is measured using a standard ruler (see Fig. 2C ).

**Fig. 3:**
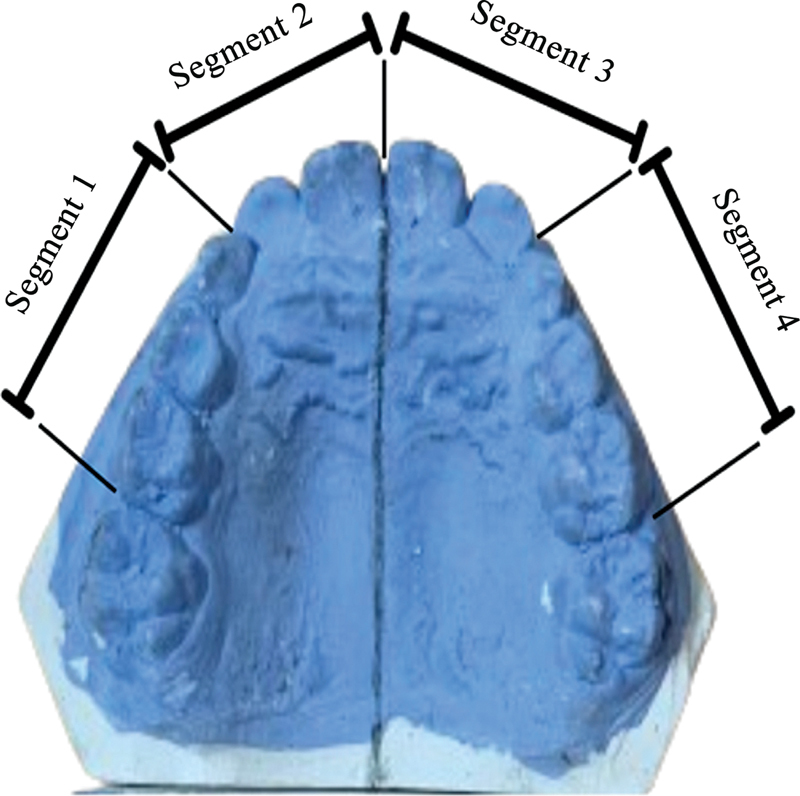
Division of the dental arch into four segments for arch length discrepancy analysis.

In the segmentation technique (see Fig. 3 ), each jaw is divided into four segments ( Fig. 3 ): M2 to C decidual; lateral to central incisor; central to lateral incisor; and C to M2 decidual. The value of each segment is summed to obtain the total available space.

### Moyers Method

In this study, a 75% probability table of Moyers method (see Table 1 ) was used to estimate the width of the permanent canine and premolar teeth based on the mesiodistal width of the lower permanent incisors. To calculate the required space, the mesiodistal width of the four permanent lower incisors is first measured, and then the value corresponding to the table for the 75% probability is added twice to the total.

**Table 1:** Moyers prediction table with 75% probability 11

**Mandibular bicuspids and cuspids**													
**Zygmondi code 21 l 12 (central and lateral incisive)**	**19.5**	**20**	**20.5**	**21**	**21.5**	**22**	**22.5**	**23**	**23.5**	**24**	**24.5**	**25**	**25.5**
Males	20.3	20.5	20.8	21	21.3	21.5	21.8	22	22.3	22.5	22.8	23	23.3
Females	19.6	19.8	20.1	20.3	20.6	20.8	21.1	21.3	21.6	21.9	22.1	22.4	22.7
**Maxillary bicuspids and cuspids**													
**21 | 12**	**19.5**	**20**	**20.5**	**21**	**21.5**	**22**	**22.5**	**23**	**23.5**	**24**	**24.5**	**25**	**25.5**
Males	20.3	20.5	20.8	21	21.3	21.5	21.8	22	22.3	22.5	22.8	23	23.3
Females	20.4	20.5	20.6	20.8	20.9	21	21.2	21.3	21.5	21.6	21.8	21.9	22.1

### Huckaba Method

The Huckaba method uses the following formula 8 :

*X* = ( *Y* ) ( *X'* )/( *Y'* ),

where *X* is the estimated size of the permanent tooth, *X'* is the radiographic size of the permanent teeth, *Y* is the size of the primary tooth on the study cast, and *Y'* is the radiographic size of the primary tooth on the study cast. To calculate the required space using the Huckaba method, the value of *X* is added to the mesiodistal width of the four lower permanent incisors. 8

**Fig. 4:**
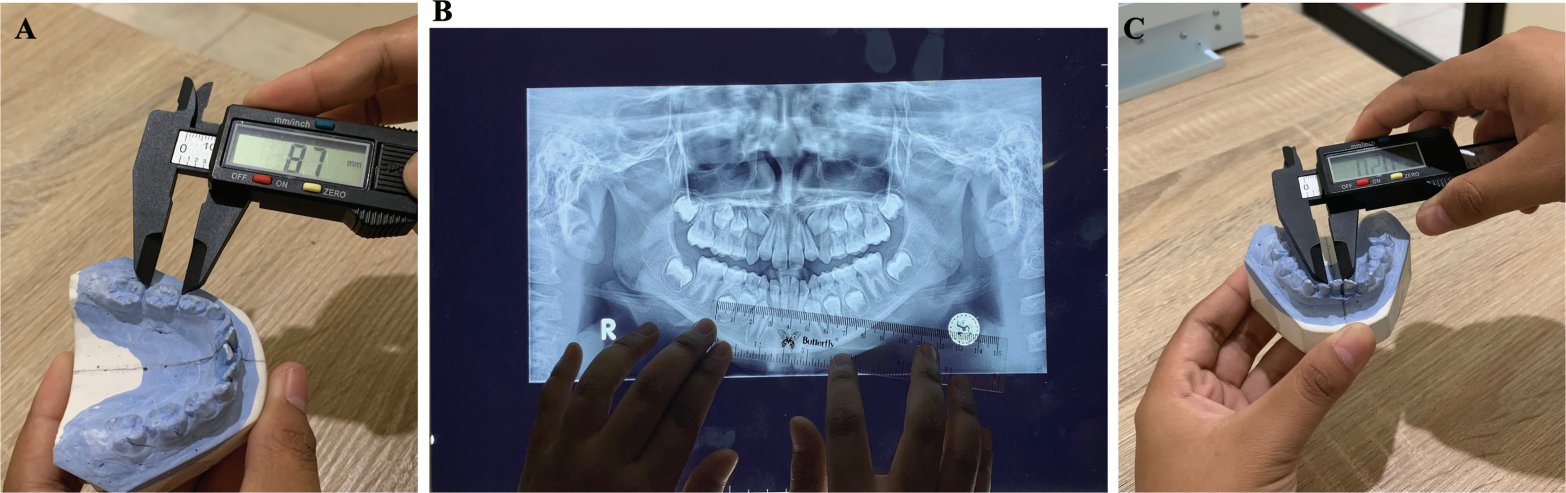
The Huckaba method. ( **A** ) Measurement of the mesiodistal width of the mandibular incisors. ( **B** ) Measurement of the mesiodistal width of the tooth bud 45 as seen on the radiograph. ( **C** ) Measurement of the mesiodistal width of tooth 45.

The first step is to calculate the mesiodistal width of the permanent canine (teeth 33 and 43), permanent first premolar (teeth 34 and 44), and permanent second premolar (teeth 35 and 45) using a ruler on the panoramic photograph. Then, add them up to get the *X* 2 value. Second, calculate the mesiodistal width of the deciduous canine (teeth 73 and 83), deciduous first molar (teeth 74 and 84), and deciduous second molar (teeth 75 and 85) using a digital caliper on the study model. Then, add them up to get the *Y* 1 value. Calculate the mesiodistal width of the deciduous canine (teeth 73 and 83), deciduous first molar (teeth 74 and 84), and deciduous second molar (teeth 75 and 85) using a ruler on the panoramic photograph (see Fig. 4A, B ). Then, add them up to get the *Y* 2 value. Calculate the value of *X* 1 with the equation for the Huckaba formula.

Add the value of *X* 1 and the value of the mesiodistal width of the permanent lateral incisor and the permanent central incisor of the lower jaw as the value of the space required (see Fig. 4C ). Measure the arch perimeter of the lower jaw using a brass wire by extending the brass wire from the mesiobuccal of the first permanent molar on one side to the mesiobuccal of the molar on the opposite side, passing through the buccal prominence and the incisal edge of the anterior teeth. The brass wire is straightened carefully and measured with a ruler to obtain the value of the arch perimeter or space available. Calculate the discrepancy of the lower jaw by subtracting the value of the space available from the value of the space required. Do the same pattern to determine the discrepancy of the upper jaw.

### Demirjian Methods

**Fig. 5:**
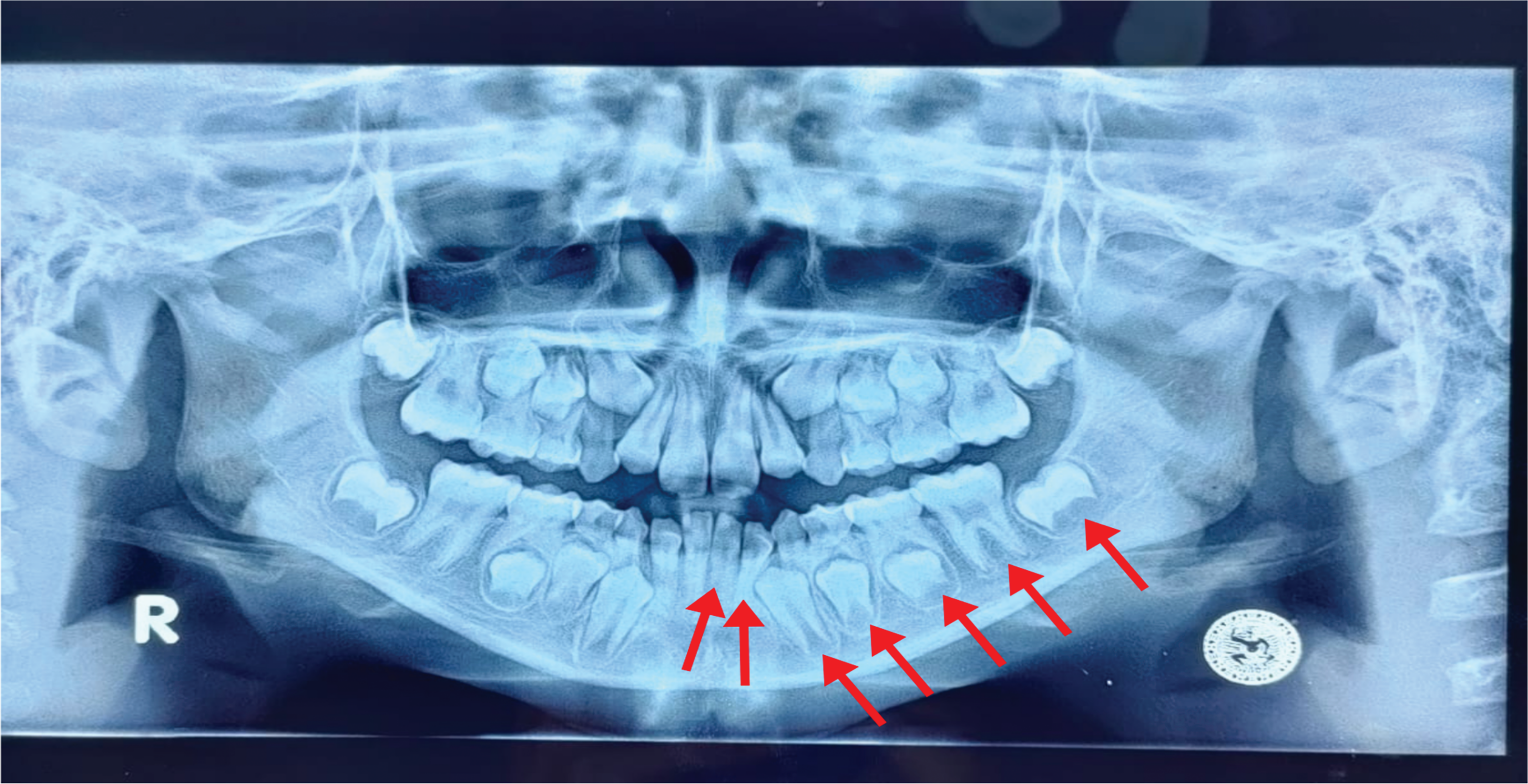
Orthopantomogram (OPG) of the teeth and permanent tooth buds in region 3 for the preparation of Huckaba's method.

Here is how the dental age analysis works using the Demirjian method. First, prepare panoramic photographs, X-ray viewers, staging tables, and Demirjian scoring tables for both women and men. Observe the radiographic images of the permanent teeth in region 3. Analyze the stages of tooth development starting from tooth 31 to teeth 32, 33, 34, 35, 36, and 37 (see Fig. 5 ). All teeth are scored on a scale of A to H according to the Demirjian atlas. Adjust to the visible radiographic images. Perform scoring according to the calcification stage of each tooth. Add up all scores and convert to the Demirjian table dental age based on each gender.

### Cervical Vertebral Maturity Stage Method

Here is how bone age analysis works based on the cervical vertebral maturity stages (CVMS).

**Fig. 6:**
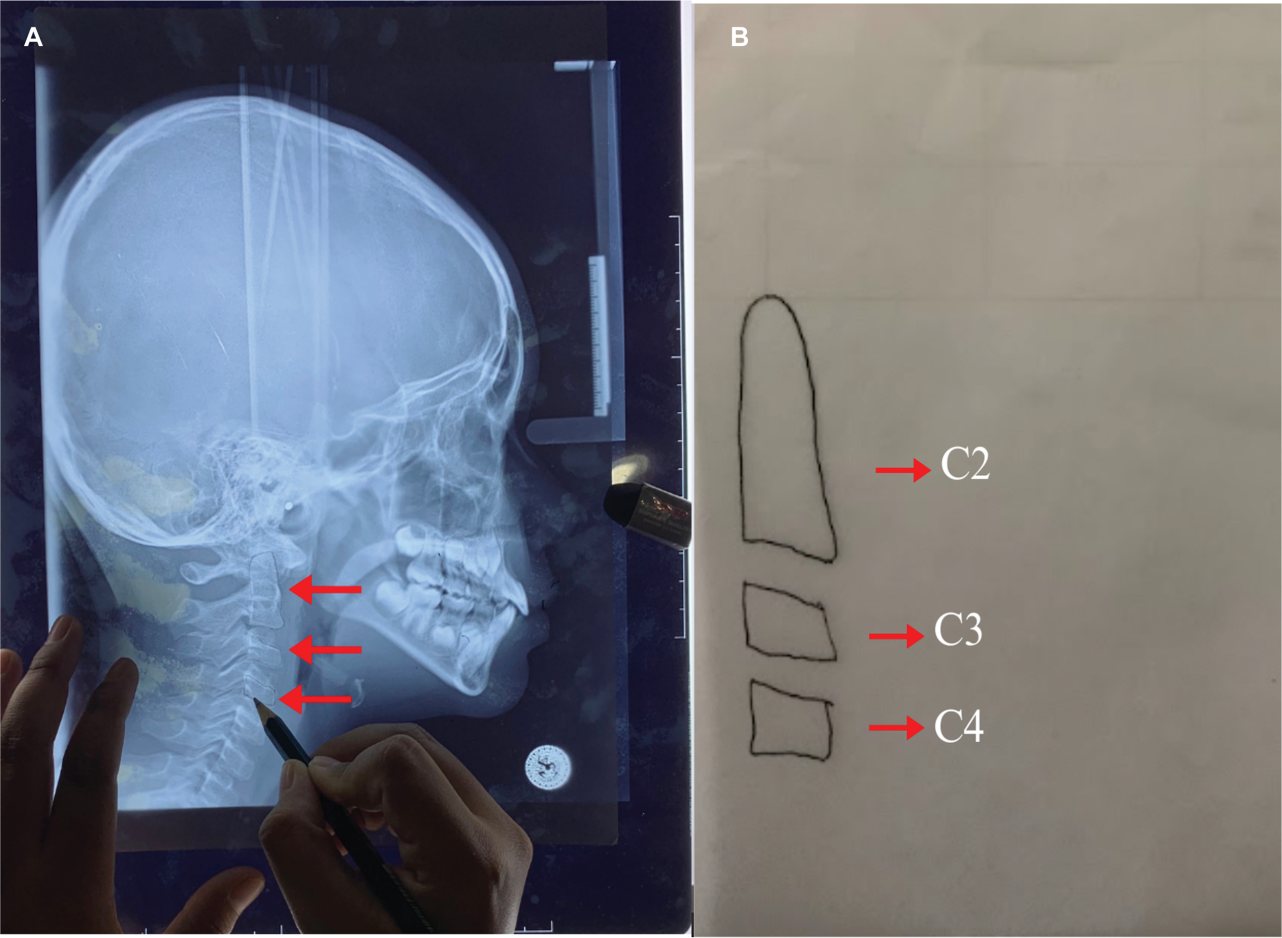
( **A** ) Tracing on tracing paper and ( **B** ) results of tracing the cervical bones C2, C3, and C4 were used to examine the bone age by means of the cervical vertebral maturity stage (CVMS).

Prepare cephalometric photographs, tracing paper, pencils, erasers, and rulers. Trace the radiographic image of the inferior border and shape of the second (C2), third (C3), and fourth (C4) cervical vertebral bodies (see Fig. 6A, B ). Convert to the CVMS characteristic table to find out the interpretation of the patient's skeletal maturity stage.

### Steiner's Cephalogram Analysis

**Fig. 7:**
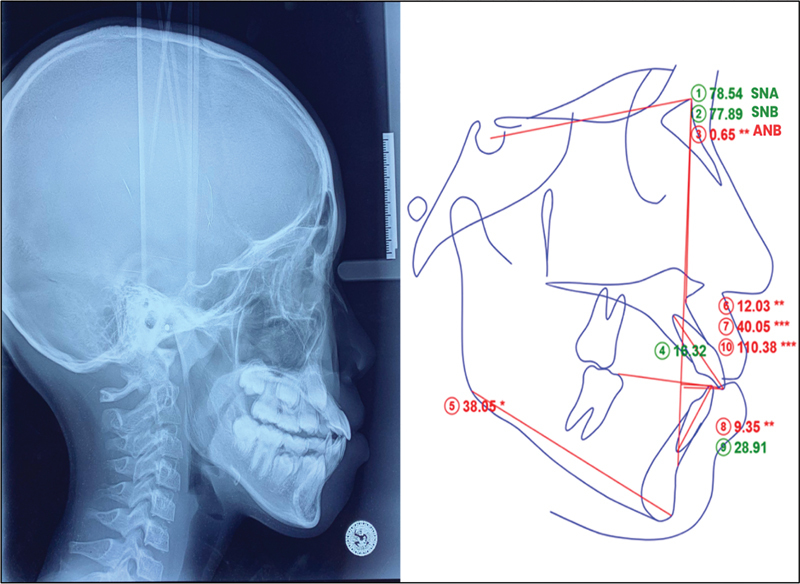
Steiner's parameters utilizing the WebCeph Automated Cephalo Tracing software ( https://webceph.com/ ).

Skeletal malocclusion was analyzed using the (Sella-Nasion-A [<SNA], Sella, Nasion, B [<SNB], [SNA-SNB] <ANB) parameters through WebCeph Automated Cephalo Tracing software ( https://webceph.com/ ; WEBCEPH 2.0.0 version, Dental Imaging Software, AssembleCircle Corp., Gyeonggi-do, Republic of Korea; see Fig. 7 ).

### Statistical Analysis

All measurements were conducted by a single operator to ensure precision and accuracy. Statistical analysis of 32 samples meeting the inclusion criteria was performed using IBM SPSS Statistics 25, with significance set at *p* < 0.05. Descriptive analysis assessed the sample distribution and frequency. The Shapiro–Wilk and Levene tests revealed non-normal distribution and lack of homogeneity, leading to the use of nonparametric Kruskal–Wallis and Mann–Whitney *U* tests for identifying significant differences.

## Results

**Fig. 8:**
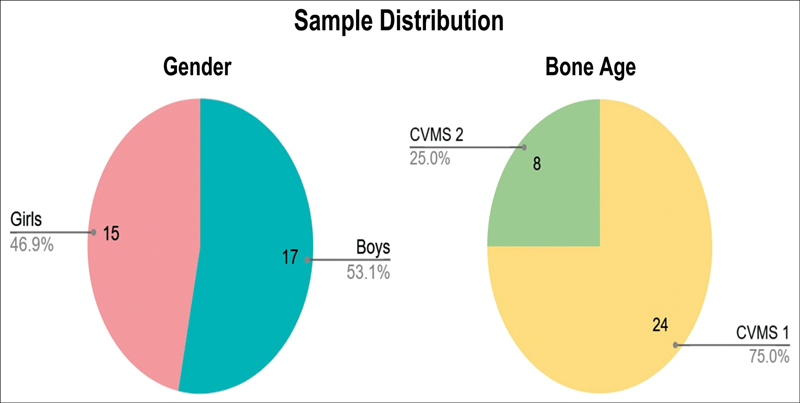
Distribution of samples by gender and bone age. CVMS, cervical vertebral maturity stage.

**Fig. 9:**
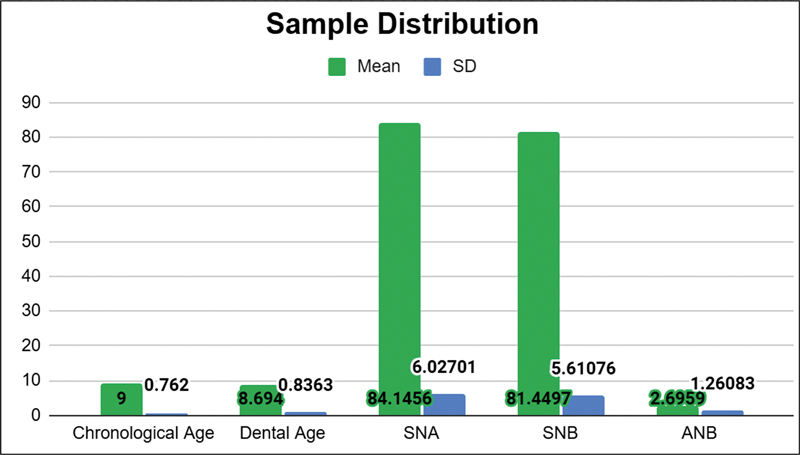
Distribution of samples by chronological age, dental age, and Steiner's analysis parameters by means of cephalometry examination. SD, standard deviation.

**Fig. 10:**
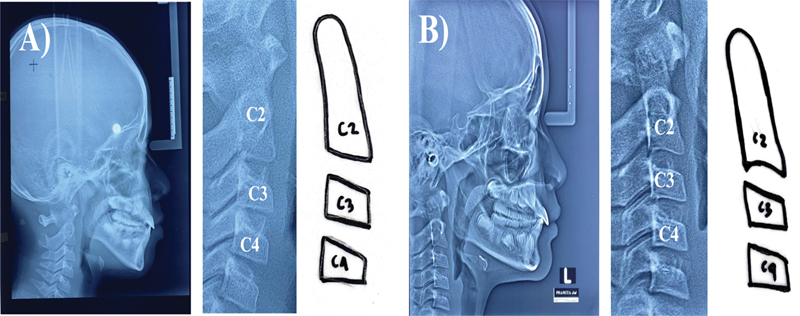
( **A** ) Cervical vertebral maturity stage (CVMS) stage 1. ( **B** ) CVMS stage 2 in the analyzed samples.

Thiry-two patients met the inclusion criteria, with most of them being males (46.9%). The average chronological age was 9 years, while the average dental age was 8.694 years. Despite some variation in both chronological and dental ages, the age range was relatively close, indicating that dental development in these samples occurred around the same time as their chronological age during the growth phase. Skeletal malocclusion was analyzed using the SNA, SNB, and ANB parameters through WebCeph Automated Cephalo Tracing software. The SNA values ranged from 74.68 to 94.78 degrees, with an average of 84.15 degrees, which is the orthognathic maxilla. The SNB values ranged from 71.69 to 91.50 degrees, with a mean of 81.45 degrees, which is the orthognathic mandible. The ANB values ranged from a minimum of 0.24 degrees to a maximum of 4.57 degrees, with an average of 2.69 degrees showing skeletal class I. All samples were classified as skeletal class I malocclusion, characterized by a straight facial profile with a harmonious relationship between the upper and lower jaws (see Table 1 , Figs. 8 and 9 ). Based on the cervical skeletal characteristics (see Fig. 10 ), the skeletal age of all samples is in the prepubertal phase with CVMS 1 and CVMS 2 (see Table 2 , Fig. 8 ).

**Table 2:** Descriptive statistical analysis of sex, bone age, chronological age, dental age, <SNA, <SNB, <ANB

Parameters	Frequency	Percentage
*Sex* Boy Girl *Bone age* CVMS 1 CVMS 2	17 15 24 8	53.1 46.9 75 25
**Parameters**	**Mean ± standard deviation**	
Chronological age Dental age SNA SNB ANB	9.00 ± 0.76 8.694 ± 0.84 84.15 ± 6.03 degrees 81.45 ± 5.6 degrees 2.7 ± 1.26 degrees	

Abbreviation: CVMS, cervical vertebral maturity stage.

**Fig. 11:**
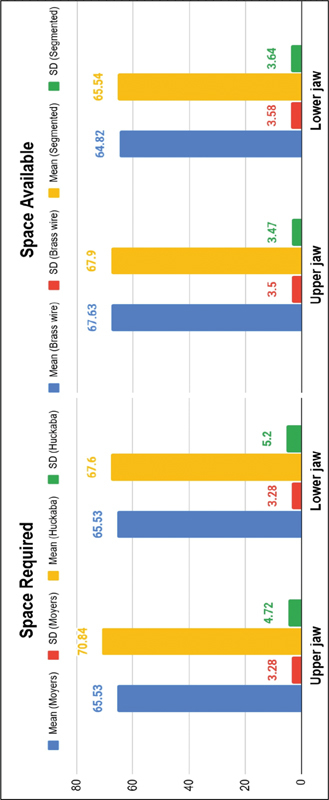
Descriptive statistical analysis of space required and space available values using the Huckaba and Moyers methods. SD, standard deviation.

In terms of space required, the Moyers method exhibits a lower mean and standard deviation than the Huckaba method in both jaws, indicating more consistent measurements. For space available, the brass wire technique in the upper jaw has a lower mean but higher variability, while in the lower jaw, it has a higher mean with lower variability compared with the segmentation technique. This highlights minor differences in the average space available between the two techniques in both jaws (see Table 3 and Fig. 11 ).

**Table 3:** Descriptive statistical analysis of space available and space required values

	*N*	Mean ± SD
**Space available** *Upper jaw* Brass wire technique Segmented technique *Lower jaw* Brass wire technique Segmented technique	32 32 32 32	67.63 ± 3.5 67.90 ± 3.47 64.82 ± 3.58 65.54 ± 3.64
**Space required** *Upper jaw* Moyers Huckaba *Lower jaw* Moyers Huckaba	32 32 32 32	65.53 ± 3.28 70.84 ± 4.72 65.53 ± 3.28 67.60 ± 5.2

Abbreviations: SD, standard deviation.

**Fig. 12:**
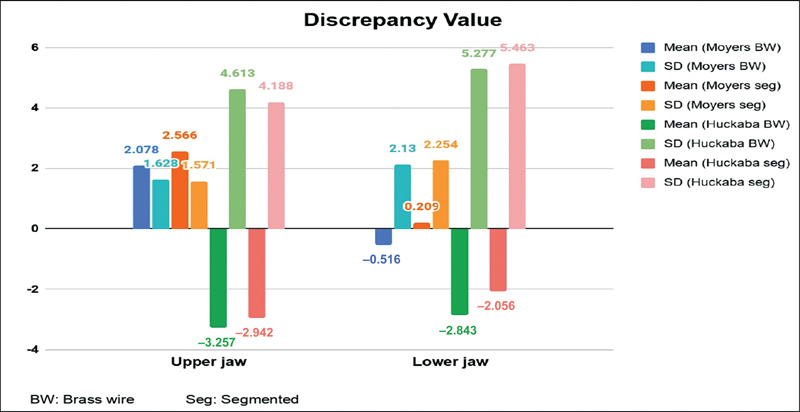
Descriptive statistical analysis of the arch length discrepancy (ALD) value in both arches using the Huckaba and Moyers methods. SD, standard deviation.

In the upper jaw, the average discrepancy value for the Moyers method was positive, whereas the Huckaba method showed a negative value. In the lower jaw, a positive average discrepancy value was observed only with the Moyers segmentation method (see Table 4 and Fig. 12 ). The Shapiro–Wilk normality test and Levene's homogeneity test results indicated that the data did not meet the assumptions of normality and homogeneity ( *p* < 0.05; see Table 5 ). The results of the Kruskal–Wallis test for discrepancy values in both jaws, using four methods, indicated a significant difference in the upper jaw ( *p* < 0.05). However, for the lower jaw, no significant difference was observed among the four methods ( *p* > 0.05; see Table 6 ). The Mann–Whitney *U* test results for the upper jaw discrepancy data revealed significant differences between the Moyers and Huckaba methods, both using the segmentation and brass wire techniques. However, no significant difference was found between the Huckaba segmented method and the Huckaba brass wire method. In contrast, for the lower jaw, a significant difference was only found between the Moyers segmented method and the Huckaba brass wire method, while no significant differences were observed in the other method comparisons (see Table 7 ).

**Table 4:** Descriptive statistical analysis of the ALD value in both arches

Methods	*N*	Mean ± SD
*Upper jaw* Moyers brass wire Moyers segmented Huckaba brass wire Huckaba segmented	32 32 32 32	2.078 ± 1.63 2.566 ± 1.57 −3.257 ± 4.61 −2.942 ± 4.19
*Lower jaw* Moyers brass wire Moyers segmented Huckaba brass wire Huckaba segmented	32 32 32 32	−0.516 ± 2.13 0.209 ± 2.25 −2.843 ± 5.28 −2.056 ± 5.46

Abbreviations: ALD, arch length discrepancy; SD, standard deviation.

**Table 5:** Results of normality and homogeneity tests of ALD examined using the Moyers and Huckaba methods

Methods	*N*	Sig. (Shapiro–Wilk)	Sig. (Levene test)
*Upper jaw* Moyers brass wire Moyers segmented Huckaba brass wire Huckaba segmented	32 32 32 32	0.01 0.01 0.14 a 0.28 a	0.01
*Lower jaw* Moyers brass wire Moyers segmented Huckaba brass wire Huckaba segmented	32 32 32 32	0.03 0.19 a 0.01 0.01	0.03

Abbreviations: ALD, arch length discrepancy.

a Information: normal and homogeneity at p > 0.05.

**Table 6:** Results of the Kruskal–Wallis test for ALD examined using the Moyers and Huckaba methods

	Sig.
Upper jaw Lower jaw	0.000 a 0.058

Abbreviations: ALD, arch length discrepancy.

a Information: significant difference at *p* < 0.05.

**Table 7:** Results of the Mann–Whitney test for ALD examined using the Moyers and Huckaba methods

Significance Value	Huckaba Brass Wire	Huckaba Segmented
Moyers brass wire		
Upper Jaw Lower Jaw	0.01 a	0.01 a
	0.08	0.56
Moyers Segmented		
Upper Jaw Lower Jaw	0.01 b	0.01 b
	0.01 b	0.12
Huckaba Segmented	0.76	–
	0.37	

Abbreviation: ALD, arch length discrepancy.

: For the upper jaw: For the lower jaw.

a,b Information: significant difference at *p* < 0.05.

## Discussion

Determining the appropriate method for analyzing ALD in MD and GDP children is a crucial step in orthodontic interceptive treatment planning. The discrepancy values obtained in this study were categorized into four groups for each jaw: the Moyers segmentation method, the Moyers brass wire method, the Huckaba brass wire method, and the Huckaba segmentation method. In the upper jaw, significant differences were found between the Huckaba and Moyers methods, both using the segmentation and brass wire techniques, while no differences were observed between the Huckaba methods. This is due to the vastly different approaches used to measure the space required in the Moyers and Huckaba methods. The Moyers method measures space required using the four permanent lower incisors and the Moyers probability table, while the Huckaba method compares the mesiodistal width of teeth on radiographs and study models. The space required measurements indicate that the Huckaba method has larger average values and standard deviations compared with the Moyers method, in both the upper and lower jaws.

The results of this study are consistent with the research conducted by Da Cruz et al in 2014 in Brazil, which demonstrated that the Huckaba method is inadequate in terms of reproducibility, particularly regarding random error. Therefore, it should be used with caution when measuring space required in the lower arch. 15 Previous research by De Oliveira et al in 2007 in Brazil indicated that the Huckaba method tends to overestimate the size of unerupted premolar and canine teeth. 16

This overestimation occurs due to the smaller distance between the primary teeth and the film compared with the permanent teeth, resulting from differences in the structure of the lingual bone. The film exposure, which touches the lingual surface of the primary teeth and the bony plate of the permanent teeth, contributes to this distance variation. 16 Similar findings were observed in a study by Flores-Mir et al in 2014, which showed that OPG tends to overestimate tooth sizes by approximately 29% compared with actual measurements taken with a digital caliper, considered the gold standard for accuracy. 17

Based on the available space values using both the segmentation and brass wire techniques, the average space available with the segmentation technique is higher than with the brass wire technique in both jaws. However, the standard deviation is smaller in the upper jaw and larger in the lower jaw. This indicates that the measurement of available space in the upper jaw has less data variation, while the lower jaw shows greater variation. In the lower jaw, the measurement of available space using the brass wire technique has the smallest average value and standard deviation. The results are influenced by the differences in the dental arch shapes, with the upper arch being more complex compared with the lower arch. According to Raberin (1993), dental arch shapes are categorized into five types: flat, pointed, mid, wide, and narrow. 18 As a result, the segmentation method is more effective in capturing subtle variations in the upper arch. In contrast, the lower arch, which is generally more symmetrical, allows the brass wire technique to yield results comparable to those obtained with segmentation.

This finding aligns with previous studies that revealed the segmentation technique is considered easier, more precise, and accurate for measuring dental arch space compared with the brass wire method, which is prone to irregularities such as crowding, rotation, or shifting of teeth. 19 However, this contrasts with a study conducted by Machado et al in 2012 in Brazil, which indicated that in terms of reproducibility, the segmentation technique was considered more accurate than the brass wire technique in the lower jaw. 20

For patients with malocclusion, determining their dental and bone ages during the GDP of interceptive orthodontics is crucial to the best possible treatment outcome. For a variety of clinical scenarios, particularly when it comes to the planning and results of treatments like orthodontic treatment, it is essential to accurately assess growth potential and the timing of growth spurts. The results of this study were consistent with earlier research showing that the variations in chronological age may correspond to the CVMS. Furthermore, the most reliable technique for determining a child's age was discovered to be the CVMS. When skeletal maturation is evaluated using the CVMS, it is possible to determine children's growth and developmental stages more precisely than with the Demirjian index. 21

This study has several limitations that warrant consideration. First, the research was confined to children with class I Angle malocclusion, thereby restricting the applicability of its findings to other malocclusion classifications, such as Angle classes II and III. Second, the Moyers probability tables employed in the Moyers method are derived from Caucasian populations, whereas this study was conducted on non-Caucasian populations, potentially compromising the validity of the space prediction outcomes. Third, the discrepancy analysis in this study did not account for the presence of proximal caries within the samples, which may have influenced the precision of the available space measurements. Additionally, the relatively limited sample size imposes further constraints on the generalizability of the study's findings.

## Conclusion

According to the study's findings, Surabayan children's class I Angle malocclusion during MD and GDP at Dental Hospital Universitas Airlangga showed significant differences in both the upper and lower arches between the Huckaba and Moyers approaches. To improve the validity and reliability of the findings, larger and more homogeneous sample populations must be used in future research.
